# Supplementary material for: Polyketide synthase gene clusters in the ericoid mycorrhizal fungus Oidiodendron maius: genome-wide prediction, expression profiling, and functional characterization of knockout mutants
Source: Front Plant Sci. 2026 Sep 16;17:1924430. doi: 10.3389/fpls.2026.1924430 (PMC13623903; doi:10.3389/fpls.2026.1924430)
Supplement: Supplementary file 1 [file SupplementaryFile1.zip › Supplementary Materials/Supplementary Folder 1.zip!/Oidma1_AssemblyScaffolds_Repeatmasked/knownclusterblast/cluster38/jgi.p_Oidma1_205481_mibig_hits.html]

| MIBiG Protein | Description | MIBiG Cluster | MiBiG Product | % ID | % Coverage | BLAST Score | E-value |
| --- | --- | --- | --- | --- | --- | --- | --- |
| EAL84395.2 | oxidoreductase,\_2OG-Fe(II)\_oxygenase\_family,\_putative | BGC0001118 | Endocrocin\_biosynthetic\_gene\_cluster | 31 | 84.0540540541 | 148 | 1.2e-35 |
| ABA70583.1 | isopenicillin\_N\_synthetase | BGC0000404 | Penicillin\_biosynthetic\_gene\_cluster | 28 | 88.6486486486 | 125 | 1.1e-28 |
| ABR12616.1 | IPN\_synthase | BGC0000405 | Penicillin\_biosynthetic\_gene\_cluster | 28 | 88.6486486486 | 125 | 1.1e-28 |
| ALI92653.1 | CitB\_Fe(II)-dependent\_oxygenase | BGC0001338 | Citrinin\_biosynthetic\_gene\_cluster | 28 | 78.9189189189 | 113 | 4.4e-25 |
| AKA59462.1 | 2OG\_Fe(II)\_oxygenase | BGC0001203 | Clarexpoxcin\_biosynthetic\_gene\_cluster | 33 | 41.6216216216 | 92 | 1.4e-18 |
